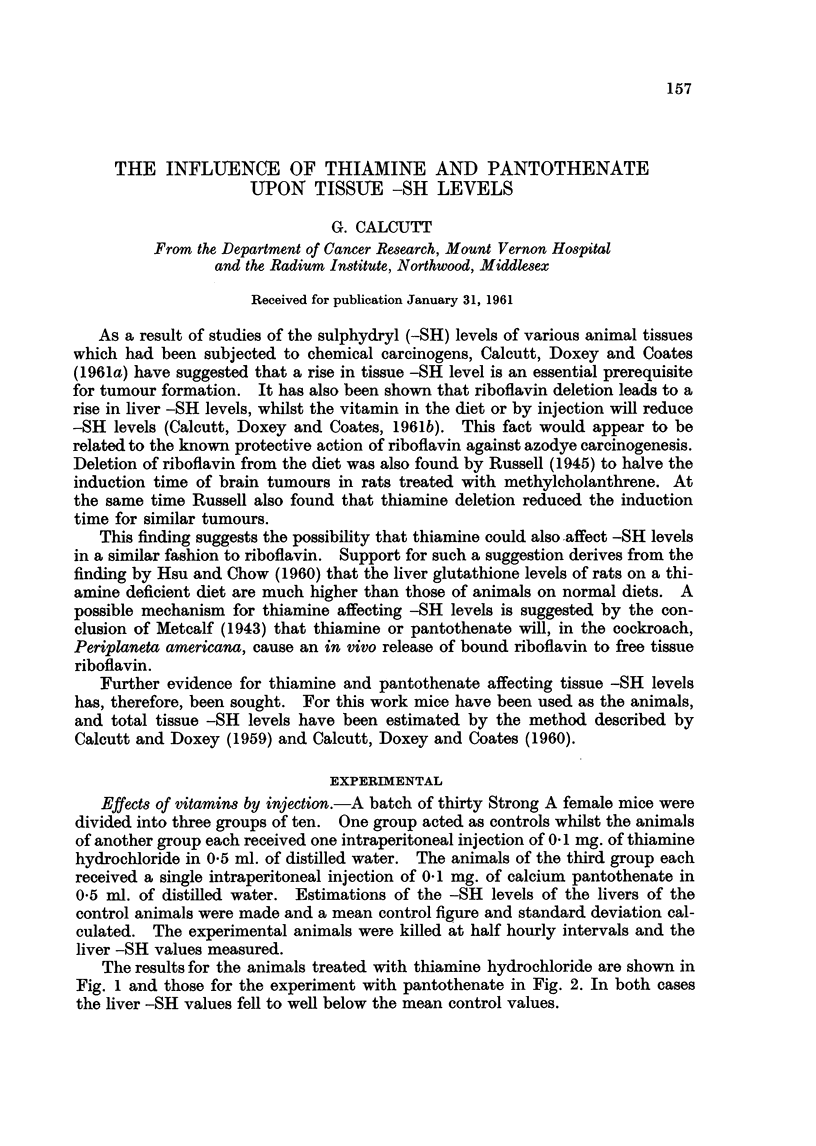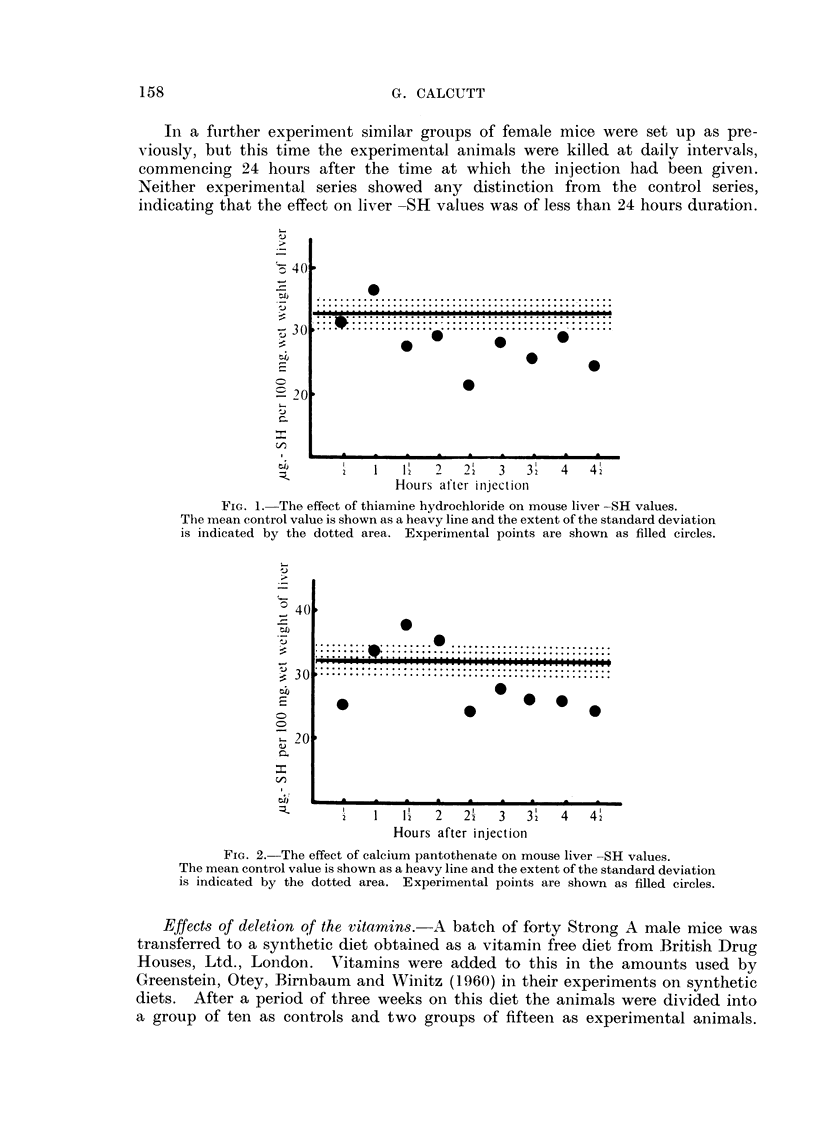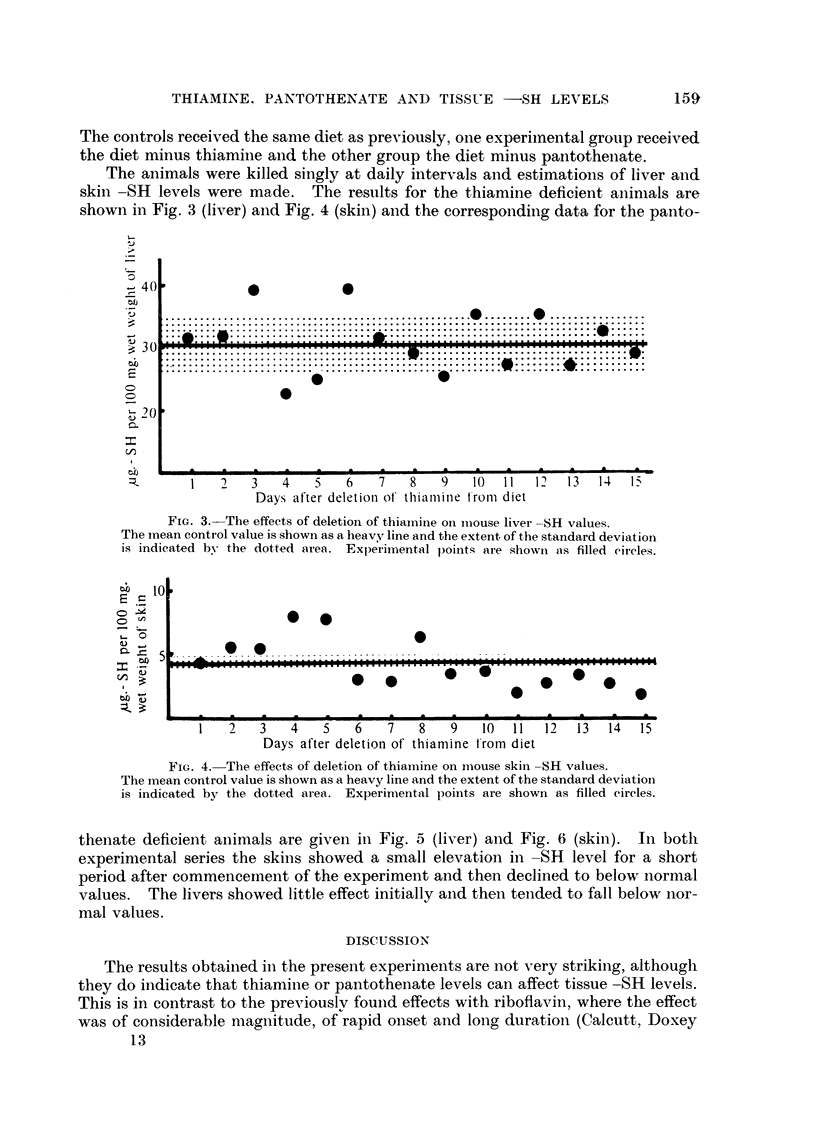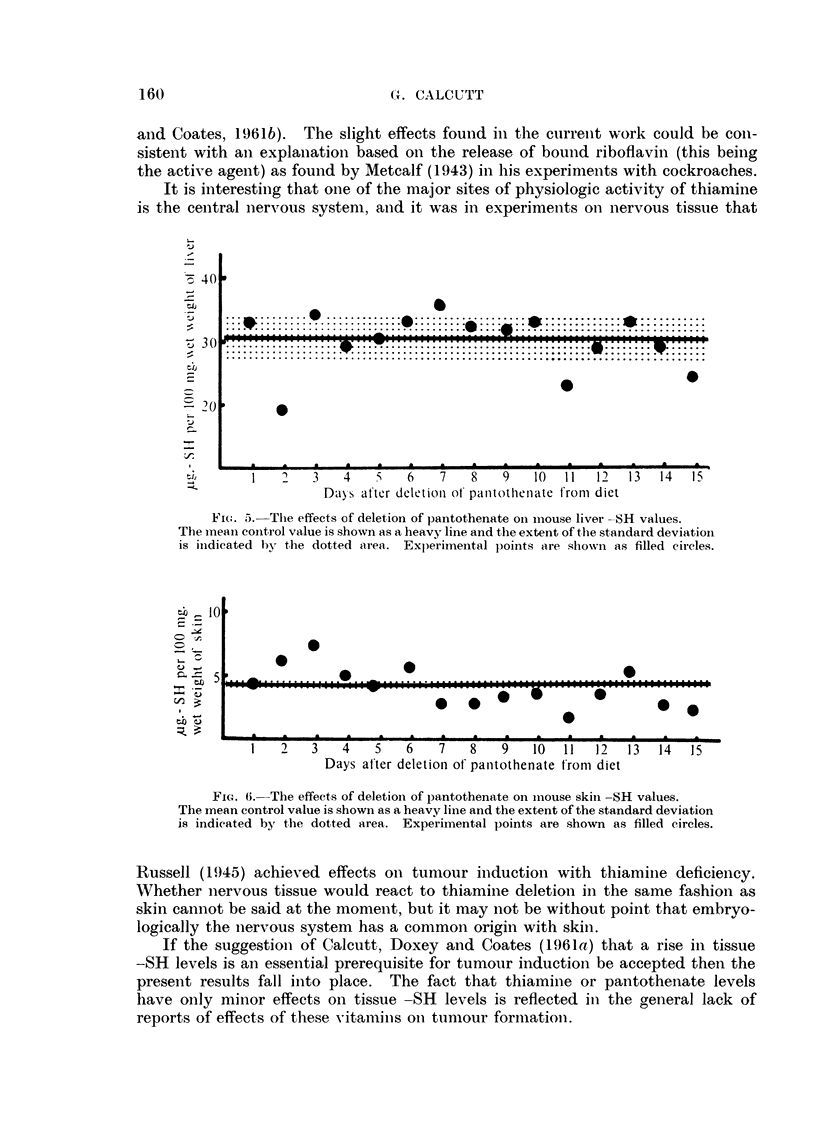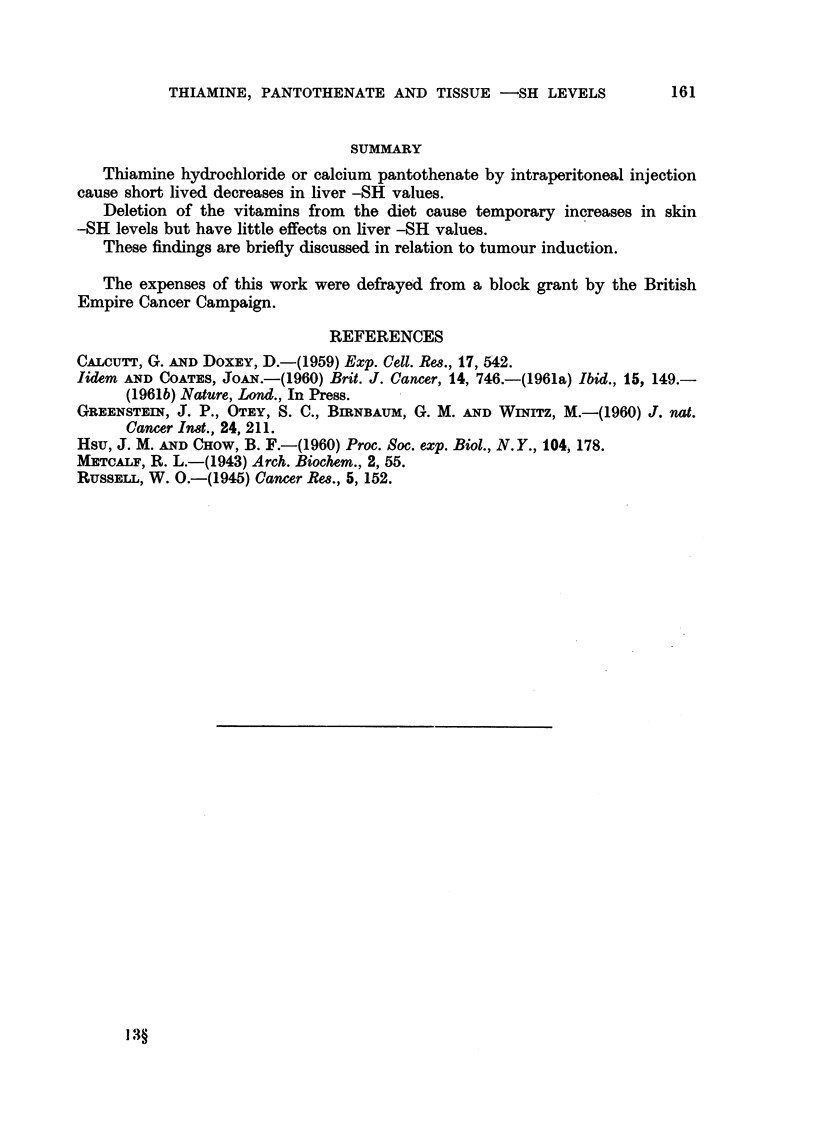# The Influence of Thiamine and Pantothenate Upon Tissue -Sh Levels

**DOI:** 10.1038/bjc.1961.20

**Published:** 1961-03

**Authors:** G. Calcutt


### THE INFLUENCE OF THIAMINE AND PANTOTHENATE UPON TISSUE –SH LEVELS

G. CALCUTT

*From the Department of Cancer Research, Mount Vernon Hospital and the Radium Institute, Northwood, Middlesex*

Received for publication January 31, 1961

As a result of studies of the sulphydryl (–SH) levels of various animal tissues which had been subjected to chemical carcinogens, Calcutt, Doxey and Coates (1961*a*) have suggested that a rise in tissue –SH level is an essential prerequisite for tumour formation. It has also been shown that riboflavin deletion leads to a rise in liver –SH levels, whilst the vitamin in the diet or by injection will reduce –SH levels (Calcutt, Doxey and Coates, 1961*b*). This fact would appear to be related to the known protective action of riboflavin against azodye carcinogenesis. Deletion of riboflavin from the diet was also found by Russell (1945) to halve the induction time of brain tumours in rats treated with methylcholanthrene. At the same time Russell also found that thiamine deletion reduced the induction time for similar tumours.

This finding suggests the possibility that thiamine could also affect –SH levels in a similar fashion to riboflavin. Support for such a suggestion derives from the finding by Hsu and Chow (1960) that the liver glutathione levels of rats on a thiamine deficient diet are much higher than those of animals on normal diets. A possible mechanism for thiamine affecting –SH levels is suggested by the conclusion of Metcalf (1943) that thiamine or pantothenate will, in the cockroach, *Periplaneta americana*, cause an *in vivo* release of bound riboflavin to free tissue riboflavin.

Further evidence for thiamine and pantothenate affecting tissue –SH levels has, therefore, been sought. For this work mice have been used as the animals, and total tissue –SH levels have been estimated by the method described by Calcutt and Doxey (1959) and Calcutt, Doxey and Coates (1960).

### EXPERIMENTAL

*Effects of vitamins by injection.*—A batch of thirty Strong A female mice were divided into three groups of ten. One group acted as controls whilst the animals of another group each received one intraperitoneal injection of 0·1 mg. of thiamine hydrochloride in 0·5 ml. of distilled water. The animals of the third group each received a single intraperitoneal injection of 0·1 mg. of calcium pantothenate in 0·5 ml. of distilled water. Estimations of the –SH levels of the livers of the control animals were made and a mean control figure and standard deviation calculated. The experimental animals were killed at half hourly intervals and the liver –SH values measured.

The results for the animals treated with thiamine hydrochloride are shown in Fig. 1 and those for the experiment with pantothenate in Fig. 2. In both cases the liver –SH values fell to well below the mean control values.

In a further experiment similar groups of female mice were set up as previously, but this time the experimental animals were killed at daily intervals, commencing 24 hours after the time at which the injection had been given. Neither experimental series showed any distinction from the control series, indicating that the effect on liver -SH values was of less than 24 hours duration.

FIG. 1.—The effect of thiamine hydrochloride on mouse liver -SH values. The mean control value is shown as a heavy line and the extent of the standard deviation is indicated by the dotted area. Experimental points are shown as filled circles.

FIG. 2.—The effect of calcium pantothenate on mouse liver -SH values. The mean control value is shown as a heavy line and the extent of the standard deviation is indicated by the dotted area. Experimental points are shown as filled circles.

*Effects of deletion of the vitamins.*—A batch of forty Strong A male mice was transferred to a synthetic diet obtained as a vitamin free diet from British Drug Houses, Ltd., London. Vitamins were added to this in the amounts used by Greenstein, Otey, Birnbaum and Winitz (1960) in their experiments on synthetic diets. After a period of three weeks on this diet the animals were divided into a group of ten as controls and two groups of fifteen as experimental animals.

The controls received the same diet as previously, one experimental group received the diet minus thiamine and the other group the diet minus pantothenate.

The animals were killed singly at daily intervals and estimations of liver and skin –SH levels were made. The results for the thiamine deficient animals are shown in Fig. 3 (liver) and Fig. 4 (skin) and the corresponding data for the panto-

Fig. 3.—The effects of deletion of thiamine on mouse liver –SH values. The mean control value is shown as a heavy line and the extent of the standard deviation is indicated by the dotted area. Experimental points are shown as filled circles.

Fig. 4.—The effects of deletion of thiamine on mouse skin –SH values. The mean control value is shown as a heavy line and the extent of the standard deviation is indicated by the dotted area. Experimental points are shown as filled circles.

thenate deficient animals are given in Fig. 5 (liver) and Fig. 6 (skin). In both experimental series the skins showed a small elevation in –SH level for a short period after commencement of the experiment and then declined to below normal values. The livers showed little effect initially and then tended to fall below normal values.

### DISCUSSION

The results obtained in the present experiments are not very striking, although they do indicate that thiamine or pantothenate levels can affect tissue –SH levels. This is in contrast to the previously found effects with riboflavin, where the effect was of considerable magnitude, of rapid onset and long duration (Calcutt, Doxey and Coates, 1961*b*). The slight effects found in the current work could be consistent with an explanation based on the release of bound riboflavin (this being the active agent) as found by Metcalf (1943) in his experiments with cockroaches.

It is interesting that one of the major sites of physiologic activity of thiamine is the central nervous system, and it was in experiments on nervous tissue that

FIG. 5.—The effects of deletion of pantothenate on mouse liver –SH values. The mean control value is shown as a heavy line and the extent of the standard deviation is indicated by the dotted area. Experimental points are shown as filled circles.

FIG. 6.—The effects of deletion of pantothenate on mouse skin –SH values. The mean control value is shown as a heavy line and the extent of the standard deviation is indicated by the dotted area. Experimental points are shown as filled circles.

Russell (1945) achieved effects on tumour induction with thiamine deficiency. Whether nervous tissue would react to thiamine deletion in the same fashion as skin cannot be said at the moment, but it may not be without point that embryologically the nervous system has a common origin with skin.

If the suggestion of Calcutt, Doxey and Coates (1961*a*) that a rise in tissue –SH levels is an essential prerequisite for tumour induction be accepted then the present results fall into place. The fact that thiamine or pantothenate levels have only minor effects on tissue –SH levels is reflected in the general lack of reports of effects of these vitamins on tumour formation.

SUMMARY

Thiamine hydrochloride or calcium pantothenate by intraperitoneal injection cause short lived decreases in liver –SH values.

Deletion of the vitamins from the diet cause temporary increases in skin –SH levels but have little effects on liver –SH values.

These findings are briefly discussed in relation to tumour induction.

The expenses of this work were defrayed from a block grant by the British Empire Cancer Campaign.

### REFERENCES

CALCUTT, G. AND DOXEY, D.—(1959) *Exp. Cell. Res.*, **17**, 542.

*Iidem* AND COATES, JOAN.—(1960) *Brit. J. Cancer*, **14**, 746.—(1961a) *Ibid.*, **15**, 149.—(1961*b*) *Nature, Lond.*, In Press.

GREENSTEIN, J. P., OTEY, S. C., BIRNBAUM, G. M. AND WINITZ, M.—(1960) *J. nat. Cancer Inst.*, **24**, 211.

HSU, J. M. AND CHOW, B. F.—(1960) *Proc. Soc. exp. Biol., N.Y.*, **104**, 178.

METCALF, R. L.—(1943) *Arch. Biochem.*, **2**, 55.

RUSSELL, W. O.—(1945) *Cancer Res.*, **5**, 152.